# Matrix Metalloproteinases in Health and Disease 2.0

**DOI:** 10.3390/biom12091190

**Published:** 2022-08-27

**Authors:** Raffaele Serra

**Affiliations:** Department of Medical and Surgical Sciences, University Magna Graecia of Catanzaro, Viale Europa, 88100 Catanzaro, Italy; rserra@unicz.it

Matrix metalloproteinases (MMPs) are members of an enzyme family that are critical for maintaining tissue allostasis. MMPs can catalyze the normal turnover of the extracellular matrix (ECM) together with other metalloproteinases (MPs) such as the ADAMs (a disintegrin and metalloproteinase) and ADAMTS (a disintegrin and metalloproteinase with thrombospondin motif) families. MMP activity is also regulated by a group of endogenous proteins, called tissue inhibitors of metalloproteinases (TIMPs). All these proteins have a pivotal role in normal physiological processes involving ECM remodeling, such as wound healing, embryogenesis, angiogenesis, bone remodeling, immunity and the female reproductive cycle. An imbalance in the expression or activity of MMPs can also have important consequences in diseases such as cancer, cardiovascular disease, peripheral vascular disease, chronic leg ulcers, multiple sclerosis and others. In the last few years, MMPs have been found to have an important role in the field of precision medicine as they may serve as biomarkers that can predict an individual’s disease predisposition, state or progression. MMPs are also thought to be a sensible target for molecular therapy [1,2,3,4,5]. The aim of this Special Issue was to explore the most recent findings in this field that may have an impact in health care systems. The article by Stewart-McGuinness at al [6], by using a modified systematic review, provided a thorough and comprehensive proteome of proteases and protease inhibitors (P/PIs) in normal and diseased human skin. The database may be used in the future to determine either which P/PIs are present in specific diseases or which diseases individual P/PIs may influence. The article by Fiotti et al. [7] investigated the association of five functional MMP polymorphisms with clinical presentation of systemic inflammatory response syndrome (SIRS), sepsis susceptibility, etiology, and survival in a cohort of patients. In particular, they found that bacteria have no association with any of the investigated MMP polymorphisms, but intracellular pathogens, viruses, and fungus likely trigger MMP expression from the host. The article by Sasaki et al. [8] explored whether cerebrospinal fluid (CSF) levels of matrix metalloproteinases (MMPs) and their inhibitors (TIMPs) were associated with brain amyloid deposition, cortical glucose metabolism, and white matter lesions (WMLs) in individuals with amnestic mild cognitive impairment (MCI). They concluded that CSF levels of MMP-2 are associated with brain amyloid deposition, and MMP-7 CSF levels are associated WMLs, suggesting that MMPs play an important role in these conditions. The review by Mougin et al. [9] focused on what is known about the ADAMTS family involved in human aneurysms from human tissues to mouse models, and they also discussed the possibility to develop, in the future, specific therapeutic approaches using inhibitors targeting the ADAMTS. Štrbac et al. [10] systematically reviewed findings on the role of MMPs as potential biomarkers and treatment targets in mesothelioma, and they found that the need for new therapeutic approaches in mesothelioma is great, and that MMPs may be interesting, not only as biomarkers, but also as treatment targets. Andreucci M et al. [11] explored the role MPs in the development of aortic aneurysms, their link with chronic kidney disease, and the mechanisms by which each of these conditions impairs the prognosis of the other. They found that MPs are centrally implicated in the pathophysiological mechanism of these conditions and play both a relevant predictive and prognostic role, and in the future, probably even an important therapeutic role. Cirillo et al. [12], through a scoping review article, explored the involvement of MMPs and ADAMs in the pathophysiology of pemphigus and pemphigoid, and they concluded that these biomarkers, and their related pathways, represent potential candidates for clinical use and for developing mechanism-based treatments of these diseases.

In conclusion, this Special Issue provides evidence that MP studies and research may be helpful in assessing the predictive and prognostic role of these biomarkers, as well as developing new related treatments aimed at improving and delivering better healthcare for a wide range of diseases.
